# Modeling the Transmission Dynamics of Clonorchiasis in Foshan, China

**DOI:** 10.1038/s41598-018-33431-w

**Published:** 2018-10-11

**Authors:** Ruixia Yuan, Jicai Huang, Xinan Zhang, Shigui Ruan

**Affiliations:** 1 School of Mathematics and Statistics, Central China Normal University, Wuhan, 430079 P. R. China; 2 Department of Mathematics, University of Miami, Coral Gables, FL 33146 USA

## Abstract

Clonorchiasis, known as the Chinese liver fluke disease, is caused by *Clonorchis sinensis* infection with food-borne liver fluke, which is transmitted via snails to freshwater fish and then to human beings or other piscivorous mammals. *Clonorchis sinensis* infection is mainly related to liver and biliary disorders, especially cholangiocarcinoma, and has an increased human-health impact due to the greater consumption of raw freshwater fish. In this article, we propose a deterministic model to describe the spread of clonorchiasis among human-snail-fish populations and use the model to simulate the data on the numbers of inspected and infected individuals of Foshan City, located in Guangdong Province in the southeast of P.R China, from 1980–2010. Mathematical and numerical analyses of the model are carried out to understand the transmission dynamics of clonorchiasis and explore effective control measures for the local outbreaks of the disease. We find that (i) the transmission of clonorchiasis from cercariae to fish plays a more important role than that from eggs to snails and from fish to humans; (ii) As the cycle of infection-treatment-reinfection continues, it is unlikely that treatment with drugs alone can control and eventually eradicate clonorchiasis. These strongly suggest that a more comprehensive approach needs to include environmental modification in order to break the cercariae-fish transmission cycle, to enhance awareness about the disease, and to improve prevention measures.

## Introduction

Clonorchiasis or Chinese liver fluke disease is a major food-borne parasitosis and caused by *Clonorchis sinensis* (*C*. *sinensis*) that parasitizes in the human intrahepatic bile duct^1,2^. It was first reported in 1875 by McConnell^3^ who observed a new species of liver fluke in the bile ducts of a patient during autopsy^4^ and the causative agent was identified as *C*. *sinensis*. Caused by the ingestion of raw or undercooked freshwater fish contaminated with the parasite *C*. *sinensis*, it is a food-borne zoonosis^5^. It is implicated in a wide spectrum of hepatobiliary diseases ranging from asymptomatic infection to more severe liver diseases including cholangitis or portal hypertension^5^. Recent evidences suggest that cholangiocarcinoma (CCA) is the most severe complication of liver fluke infection and *C*. *sinensis* infection is classified as “carcinogenic to humans” by the International Agency for Research on Cancer (IARC) in 2009^6^. Meta-analysis and systematic reviews show pooled odds ratios for *C*. *sinensis* infection and cholangiocarcinoma ranging between 4.5 and 6.1^7,8^. It was estimated that more than 601 million people were at the risk of *C*. *sinensis* infection and at least 35 million cases of clonorchiasis worldwide, contributing to approximately 5600 deaths in 2005^5,9^. The overwhelming majority of clonorchiasis cases occur in endemic areas in eastern Asia, including Korea Peninsula, Japan, China, etc.^7,8,10^. Particularly, China has the biggest share with an estimated 13 million people infected with clonorchiasis^3^. Zhou *et al*.^11^ reported that the trend of infection risk is increasing from 2005 onwards and resulted in a threat to the public health in epidemic regions^12,13^. Specially, they estimated that around 14.8 million people in China were infected with *C*. *sinensis* in 2010^11^ and there are two major endemic regions in China: provinces in the northeast such as Heilongjiang and Jilin; and provinces in the southeast including Guangdong and Guangxi^8,14^.

*C*. *sinensis* is characterised by an alternation of sexual and asexual reproduction in different hosts^15,16^, involving three intermediate hosts including freshwater snails (act as the first intermediate hosts), occasionally shrimps and freshwater fish (act as the second intermediate hosts), and humans or carnivorous mammals (act as the definitive hosts)^3,5,17^. Simply speaking, eggs laid by hermaphroditic adult worms reach the intestine with bile fluids and are eliminated with the faeces^18^. Subsequently, freshwater snails swallow the eggs^19^, through asexual reproduction, sporocysts, rediae, and then cercariae are produced. After escaping from the snails, cercariae then infect and adhere to freshwater fish^20^ and develop into mature metacercariae. When people or other piscivorous mammals eat insufficiently cooked or raw infected fish, they will become the definitive hosts^3,9^. Patients with low infection intensity are often show only mild symptoms or asymptomatic or even without any performance, whereas patients with high infection intensity often show unspecific symptoms, such as indigestion, asthenia, nausea, vertigo, dizziness, headache, abdominal discomfort, abdominal pain, or diarrhoea, especially in the right upper quadrant^3^. Typical physical signs of *C*. *sinensis* infection are liver tenderness, jaundice and hepatomegaly^3^.

In recent years, clonorchiasis has been studied from many different perspectives, including epidemiological features^3^, key clinica, geography, diagnostic, immunology, etc. Qian *et al*.^10^ presented comparisons between clonorchiasis and hepatitis B in terms of carcinogenicity, disability and epidemiology, clinical symptoms as well as changing trends. Lai *et al*.^11^ carried out Bayesian variable selections to identify the most important predictors of *C*. *sinensis* risk and their results provide spatially relevant information for guiding clonorchiasis control interventions in China. Specially, researchers have obtained some protective effects about vaccine, but only in rat models^21–23^. Though clonorchiasis has been studied for more than 140 years and we have a sound understanding of clonorchiasis, but there has been no study using mathematical modelling approach to assess different tools and strategies for the control of clonorchiasis. However, many researchers have studied vector-borne diseases that have only one main intermediate host^24–28^. The results in^25,26^ showed that control strategies that target on the transmission of schistosomiasis from the snail to man will be more effective than those that block the transmission from man to snail. Particularly, Chiyaka *et al*.^25^ constructed a deterministic mathematical model of schistosomiasis where the miracidia and cercariae dynamics are incorporated.

To understand the transmission dynamics of clonorchiasis and to explore effective control and prevention measures, in this paper we propose a deterministic model for the human-snail-fish transmission of clonorchiasis. The aim is to use mathematical modeling approach to gain some insights into the transmission dynamics of clonorchiasis in these populations. The model is a system described by ten ordinary differential equations counting for susceptible and infected human, snail, fish subpopulations, recovered people, exposed people, egg and cercaria. We study the basic properties of the model, including the boundedness of solutions, existence and stability of the disease-free equilibrium and the endemic equilibrium. Then, to validate the model, we use the model to simulate the data on the numbers of inspected and infected individuals in Foshan City, Guangdong Province, China, from 1980 to 2010. Specially, it should be pointed out that we regard the number of inspected persons as the number of exposed individuals. Numerical simulations match the data reasonably well. We also give some reasonable predictions for Foshan City for the coming years. Finally, by carrying out sensitivity analysis of the basic reproduction number *R*_0_ in terms of model parameters, we try to explore some strategies to prevent and control the local infection of clonorchiasis.

The remaining part of this paper is organized as follows. In section 2, we formulate a mathematical model to describe the spread of clonorchiasis among snail, fish and human populations. We calculate the basic reproductive number of the model, discuss the global stability of the disease-free equilibrium and the endemic equilibrium in Section 3. Data simulations and sensitivity analysis of *R*_0_ on model parameters are carried out in Section 4. A brief discussion and various control measures are given in Section 5.

## Methods

In this section, we present a mathematical model to study the transmission dynamics of clonorchiasis among human, snail and fish populations. The model is based on a susceptible, exposed, infectious, and recovered (SEIR) structure and explains the transmission process among humans, snails and fish.

### The Model

**Figure 1:**
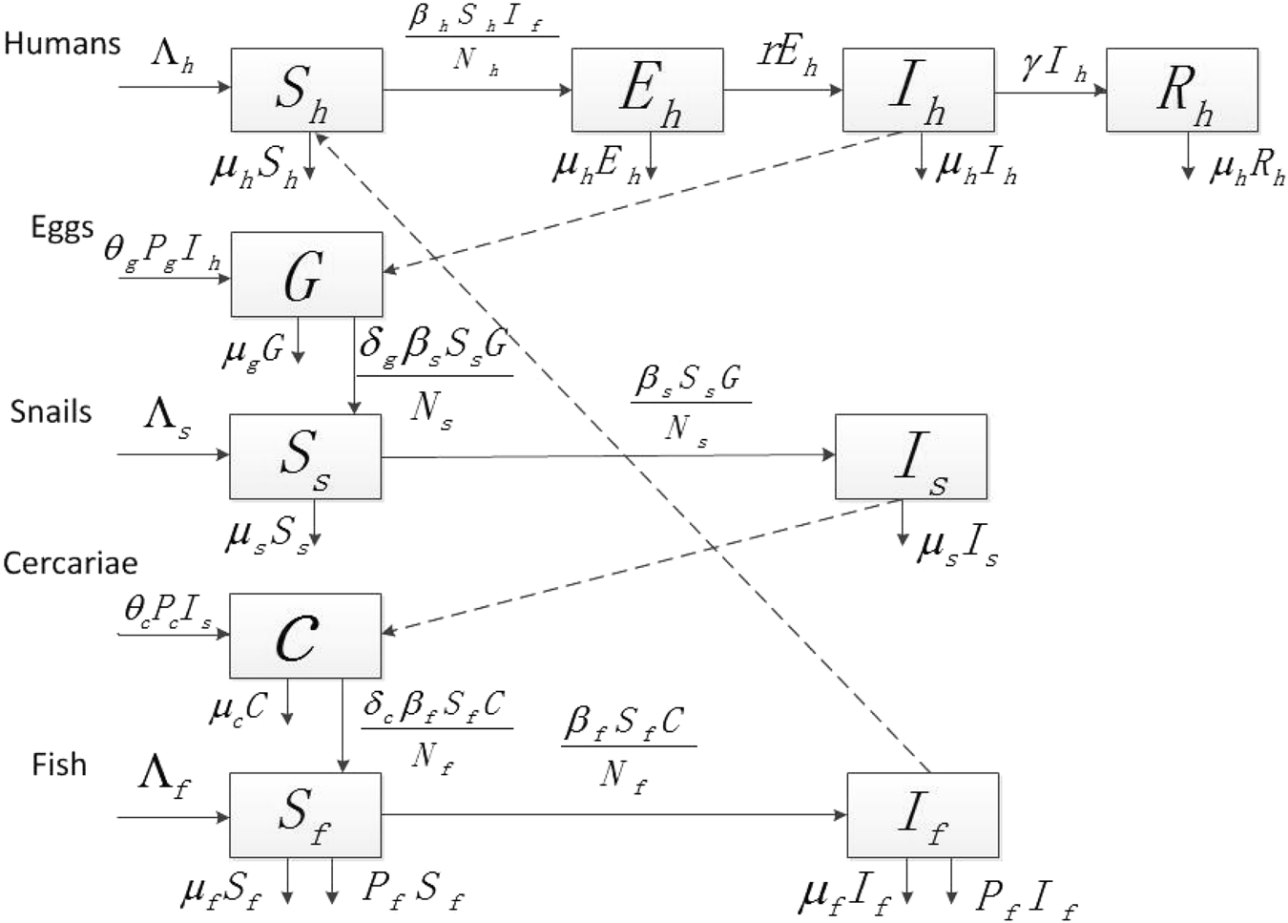
Flowchart of the clonorchiasis model for the transmission of clonorchiasis among human, snail and fish populations.

Let *S*_*h*_(*t*), *E*_*h*_(*t*), *I*_*h*_(*t*) and *R*_*h*_(*t*) denote the number of susceptible, exposed, infectious, and recovered humans at time *t*, respectively. Similarly, *S*_*s*_(*t*), *I*_*s*_(*t*), *S*_*f*_(*t*) and *I*_*f*_(*t*) represent the number of susceptible and infectious snails/fish at time *t*, respectively. Let *G*(*t*) and *C*(*t*) be the population of eggs and cercariae, respectively. Here the total human population is denoted by *N*_*h*_(*t*) = *S*_*h*_(*t*) + *E*_*h*_(*t*) + *I*_*h*_(*t*) + *R*_*h*_(*t*). Meanwhile, *N*_*s*_(*t*) = *S*_*s*_(*t*) + *I*_*s*_(*t*) and *N*_*f*_(*t*) = *S*_*f*_(*t*) + *I*_*f*_(*t*) are the total numbers of snails and fish. Our assumptions are given in the flowchart (Fig. 1).

Considering an infected individual, a portion *P*_*g*_ of eggs leave the infectious body with the faeces or urine and at a rate of *θ*_*g*_ find their way into the fresh water. The infected snails will then release a second form of free swimming larva called a cercaria, a portion *P*_*c*_, at a rate *θ*_*c*_.

For other parameters, those Λ_*i*_(*i* = *h*, *s*, *f*) are the recruitment rates of humans, snails and fish, respectively. *β*_*i*_(*i* = *h*, *s*, *f*) are the transmission rates of clonorchiasis from fish to humans, eggs to snails, and cercariae to fish. *δ*_*g*_ and *δ*_*c*_ refer to as per consumption coefficient of the eggs by snails and those of the cercariae by fish, respectively. *γ* describes the recovery rate. $\frac{1}{r}$ is the average period of latency. All those labelled *μ*_*i*_(*i* = *h*, *s*, *f*, *g*, *c*) are defined as the natural death rates of humans, snails, fish, eggs and cercariae. The predation rate of fish is *p*.

The number of eggs consumed by snails compared to the number of eggs in the environment is very small. Thus, the deletion $\frac{{\delta }_{g}{\beta }_{s}{S}_{s}(t)G(t)}{{N}_{s}(t)}$ by snails from the egg population can be ignored. Similarly, the deletion $\frac{{\delta }_{c}{\beta }_{f}{S}_{f}(t)C(t)}{{N}_{f}(t)}$ by fish from the cercaria population can be ignored, too.

Based on the assumptions and the flowchart, our model is consisted of the following equations:

1 $$\{\begin{array}{rcl}{S}_{h}^{\text{'}}(t) & = & {{\rm{\Lambda }}}_{h}-\frac{{\beta }_{h}{S}_{h}(t){I}_{f}(t)}{{N}_{h}(t)}-{\mu }_{h}{S}_{h}(t),\\ {E}_{h}^{^{\prime} }(t) & = & \frac{{\beta }_{h}{S}_{h}(t){I}_{f}(t)}{{N}_{h}(t)}-r{E}_{h}(t)-{\mu }_{h}{E}_{h}(t),\\ {I}_{h}^{^{\prime} }(t) & = & r{E}_{h}(t)-\gamma {I}_{h}(t)-{\mu }_{h}{I}_{h}(t),\\ {R}_{h}^{^{\prime} }(t) & = & \gamma {I}_{h}(t)-{\mu }_{h}{R}_{h}(t),\\ G^{\prime} (t) & = & {\theta }_{g}{P}_{g}{I}_{h}(t)-{\mu }_{g}G(t),\\ {S}_{s}^{^{\prime} }(t) & = & {{\rm{\Lambda }}}_{s}-\frac{{\beta }_{s}{S}_{s}(t)G(t)}{{N}_{s}(t)}-{\mu }_{s}{S}_{s}(t),\\ {I}_{s}^{^{\prime} }(t) & = & \frac{{\beta }_{s}{S}_{s}(t)G(t)}{{N}_{s}(t)}-{\mu }_{s}{I}_{s}(t),\\ C^{\prime} (t) & = & {\theta }_{c}{P}_{c}{I}_{s}(t)-{\mu }_{c}C(t),\\ {S}_{f}^{^{\prime} }(t) & = & {{\rm{\Lambda }}}_{f}-\frac{{\beta }_{f}{S}_{f}(t)C(t)}{{N}_{f}(t)}-p{S}_{f}(t)-{\mu }_{f}{S}_{f}(t),\\ {I}_{f}^{^{\prime} }(t) & = & \frac{{\beta }_{f}{S}_{f}(t)C(t)}{{N}_{f}(t)}-p{I}_{f}(t)-{\mu }_{f}{I}_{f}(t)\end{array}$$

under the initial value conditions *S*_*h*_(0) ≥ 0, *E*_*h*_(0) ≥ 0, *I*_*h*_(0) ≥ 0, *R*_*h*_(0) ≥ 0, *G*(0) ≥ 0, *S*_*s*_(0) ≥ 0, *I*_*s*_(0) ≥ 0, *C*(0) ≥ 0, *S*_*f*_(0) ≥ 0, *I*_*f*_(0) ≥ 0. All parameters are nonnegative constants with their biological interpretations given in Table 1.

**Table 1:** Description of model parameters (PRM) and their values (unit: *year*^−1^).

PRM	Value	Interpretation	Source
Λ_*h*_	5 × 10^4^	Recruitment rate of susceptible humans	fitting
Λ_*s*_	3.12 × 10^6^	Recruitment rate of susceptible snails	fitting
Λ_*f*_	1 × 10^3^	Recruitment rate of susceptible fish	fitting
*β*_*h*_	9.69 × 10^−2^	Transmission rate from infected fish to human	fitting
*β*_*s*_	5.54 × 10^−4^	Transmission rate from egg to snail	fitting
*β*_*f*_	3.59 × 10^−3^	Transmission rate from cercaria to fish	fitting
*μ*_*h*_	1.4 × 10^−2^	Death rate of human hosts	^26^
*μ*_*s*_	1	Death rate of snails	^24, 31^
*μ*_*f*_ + *p*	0.3031	Death rate and predation rate of fish	fitting
*μ*_*g*_	3.85 × 10^−2^	Death rate of eggs	^3^
*μ*_*c*_	2.614	Death rate of cercariae	^3^
*P*_*c*_	452	Number of cercariae in every infected snail	fitting
*P*_*g*_	1.46 × 10^6^	Number of embryonated eggs passed by each infected human	^3^
*θ*_*g*_	1 × 10^−2^	Rate of eggs into the fresh water (snail)	fitting
*θ*_*c*_	0.1564	Rate of cercariae released from infected snails	fitting
*γ*	0.73	Per capita recovery rate of human hosts	^32^
*r*	0.2405	Transmission rate from exposed to infectious human	fitting

Specific parameter values will be given in section 4 when the model is used to fit the data of inspected and infected individuals of Foshan City from^29^. Notice that the clonorchiasis data reported by^29^ are annual data. In order to use model (1) to simulate the annual clonorchiasis data from^29^, we use a percentage per year to describe some parameters so that the time unit is year. For example, *μ*_*s*_ = 1/year means that the average life of snails is 12 months.

### The Basic Reproduction Number

Each of the total subpopulations *N*_*h*_(*t*), *N*_*s*_(*t*), *N*_*f*_(*t*), *G*(*t*) and *C*(*t*) is assumed to be nonnegative at *t* = 0. Using standard analysis we know that all solutions to system (1) are nonnegative. The region

$$\begin{array}{rcl}{\rm{\Omega }} & = & \{({S}_{h}(t),{E}_{h}(t),{I}_{h}(t),{R}_{h}(t),G(t),{S}_{s}(t),{I}_{s}(t),C(t),{S}_{f}(t),{I}_{f}(t))\\  &  & \in \,{{\mathbb{R}}}_{+}^{10}|0\le {S}_{h}(t)+{E}_{h}(t)+{I}_{h}(t)+{R}_{h}(t)\le \frac{{{\rm{\Lambda }}}_{h}}{{\mu }_{h}},\,0\le {S}_{s}(t)\\  &  & +\,{I}_{s}(t)\le \frac{{{\rm{\Lambda }}}_{s}}{{\mu }_{s}},0\le {S}_{f}(t)+{I}_{f}(t)\le \frac{{{\rm{\Lambda }}}_{f}}{p+{\mu }_{f}},\,0\le G(t)\\  &  & \le \,\frac{{\theta }_{g}{P}_{g}{{\rm{\Lambda }}}_{h}}{{\mu }_{g}{\mu }_{h}},\,0\le C(t)\le \frac{{\theta }_{c}{P}_{c}{{\rm{\Lambda }}}_{s}}{{\mu }_{c}{\mu }_{s}}\},\end{array}$$

is positively invariant for system (1).

Model (1) has a disease-free equilibrium given by

$${E}^{0}=(\frac{{{\rm{\Lambda }}}_{h}}{{\mu }_{h}},0,0,0,0,\frac{{{\rm{\Lambda }}}_{s}}{{\mu }_{s}},0,0,\frac{{{\rm{\Lambda }}}_{f}}{p+{\mu }_{f}},0).$$

Following the methods and results in Diekmann *et al.*^18^ and van den Driessche and Watmough^30^, we define the basic reproduction number as

2 $${R}_{0}=\sqrt[3]{\frac{{\beta }_{h}{\beta }_{s}{\beta }_{f}{\theta }_{c}{P}_{c}{\theta }_{g}{P}_{g}r}{{\mu }_{s}(p+{\mu }_{f}){\mu }_{c}{\mu }_{g}({\mu }_{h}+\gamma )\,(r+{\mu }_{h})}}.$$

Moreover, if *R*_0_ < 1 the disease-free equilibrium *E*^0^ of system (1) is locally asymptotically stable; if *R*_0_ > 1 then *E*^0^ is unstable and a positive endemic equilibrium

$${E}^{\ast }=({S}_{h}^{\ast },{E}_{h}^{\ast },{I}_{h}^{\ast },{R}_{h}^{\ast },{G}^{\ast },{S}_{s}^{\ast },{I}_{s}^{\ast },{C}^{\ast },{S}_{f}^{\ast },{I}_{f}^{\ast })$$

exists, where

$$\begin{array}{l}{I}_{f}^{\ast }=\tfrac{{\mu }_{s}{\mu }_{g}{\mu }_{c}{{\rm{\Lambda }}}_{h}{{\rm{\Lambda }}}_{s}{{\rm{\Lambda }}}_{f}(\gamma +{\mu }_{h})\,(r+{\mu }_{h})({R}_{0}^{3}-1)}{{\mu }_{s}{\mu }_{g}{\mu }_{c}{\beta }_{h}{{\rm{\Lambda }}}_{s}{{\rm{\Lambda }}}_{f}(\gamma +{\mu }_{h})\,(r+{\mu }_{h})+{\theta }_{g}{P}_{g}{{\rm{\Lambda }}}_{h}r{\beta }_{h}{\beta }_{s}({\beta }_{f}{{\rm{\Lambda }}}_{s}{\theta }_{c}{P}_{c}+{\mu }_{s}{\mu }_{c}{{\rm{\Lambda }}}_{f})},\,{C}^{\ast }=\tfrac{(p+{\mu }_{f}){{\rm{\Lambda }}}_{f}{I}_{f}^{\ast }}{{\beta }_{f}({{\rm{\Lambda }}}_{f}-(p+{\mu }_{f}){I}_{f}^{\ast })},{S}_{h}^{\ast }=\tfrac{{{\rm{\Lambda }}}_{h}^{2}}{{\mu }_{h}{{\rm{\Lambda }}}_{h}+{\mu }_{h}{\beta }_{h}{I}_{f}^{\ast }},\\ {E}_{h}^{\ast }=\tfrac{{\mu }_{h}{\beta }_{h}{I}_{f}^{\ast }{S}_{h}^{\ast }}{{{\rm{\Lambda }}}_{h}(r+{\mu }_{h})},\,{I}_{s}^{\ast }=\tfrac{{\mu }_{c}(p+{\mu }_{f}){{\rm{\Lambda }}}_{f}{I}_{f}^{\ast }}{{\theta }_{c}{P}_{c}{\beta }_{f}({{\rm{\Lambda }}}_{f}-(p+{\mu }_{f}){I}_{f}^{\ast })},\,{N}_{f}^{\ast }=\tfrac{{{\rm{\Lambda }}}_{f}}{(p+{\mu }_{f})},\,{N}_{h}^{\ast }=\tfrac{{{\rm{\Lambda }}}_{h}}{{\mu }_{h}},{I}_{h}^{\ast }\\ \begin{array}{c}\,={N}_{h}^{\ast }-({S}_{h}^{\ast }+{R}_{h}^{\ast }+{E}_{h}^{\ast }),\,{R}_{h}^{\ast }=\tfrac{\gamma }{{\mu }_{h}}{I}_{h}^{\ast },\,{G}^{\ast }=\tfrac{{\mu }_{s}{{\rm{\Lambda }}}_{s}{I}_{s}^{\ast }}{{\beta }_{s}({{\rm{\Lambda }}}_{s}-{\mu }_{s}{I}_{s}^{\ast })},\,{S}_{f}^{\ast }\\ \,={N}_{f}^{\ast }-{I}_{f}^{\ast },\,{N}_{s}^{\ast }=\tfrac{{{\rm{\Lambda }}}_{s}}{{\mu }_{s}},\,{S}_{s}^{\ast }={N}_{s}^{\ast }-{I}_{s}^{\ast }.\end{array}\end{array}$$

Furthermore, if *R*_0_ > 1 the endemic equilibrium E^*^ of system (1) is locally asymptotically stable in the region Ω. The statements and proofs of these results are given in the Electronic Supplementary Material.

## Results

### Data from Foshan City

**Figure 2:**
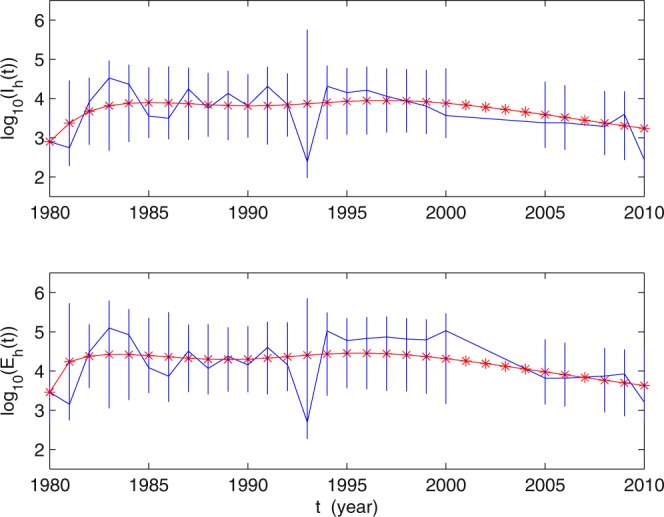
The solid blue curves represent the values of log_10_ (*E*(*t*)) and log_10_ (*I*(*t*)), where the data of *E*(*t*) and *I*(*t*) are reported in^29^. The solid red curves are simulated by using the model (1), the vertical segments are shown the 95% confidence intervals of the values of log_10_ (*E*(*t*)) and log_10_ (*I*(*t*)). The values of parameters are given in Table 1. The initial values used in the simulations are given in Table 3.

Foshan City in Guangdong Province, China, was selected as the simulating area, based on the following reasons. First, Guangdong Province, extending from the Pearl and Han rivers, has the highest prevalence of *C*. *sinensis*^9^. Second, Foshan City ranks among the top infection areas in Guangdong due to the special diet habits of local people^29^. Third, some villages of Foshan City (Shibo in Shunde district) have not yet received mass drug administration^22^. In this section, we first use model (1) to simulate the data on the numbers of inspected and infected humans of Foshan City from 1980 to 2010 provided by^29^. The numbers of inspected and infected individuals are of the order of magnitude of 1 × 10^2^ to 1 × 10^6^, which is uneasy to do numerical fitting. So we turn the data into a base 10 logarithm. In other words, we substitute log_10_ (*E*_*h*_(*t*)) and log_10_ (*I*_*h*_(*t*)) for the numbers of inspected and infectious of humans. From 1980–2010, the values of log_10_ (*E*_*h*_(*t*)) and log_10_ (*I*_*h*_(*t*)) are shown in Table 2, where ‘−’ means that there is no survey data in that year. Numerical simulations of log_10_ (*E*_*h*_(*t*)) and log_10_ (*I*_*h*_(*t*)) are shown in Fig. 2.

**Table 2:** The values of log_10_ (*E*(*t*)) and log_10_ (*I*(*t*)).

Year	log_10_ (*E*(*t*))	log_10_ (*I*(*t*))	Year	log_10_ (*E*(*t*))	log_10_ (*I*(*t*))	Year	log_10_ (*E*(*t*))	log_10_ (*I*(*t*))
1980	3.4597	2.8976	1991	4.5999	4.3089	2002	—	—
1981	3.1511	2.7412	1992	4.158	3.8479	2003	—	—
1982	4.4816	3.9132	1993	2.6972	2.3909	2004	—	—
1983	5.0955	4.5209	1994	5.0207	4.3112	2005	3.8152	3.378
1984	4.9212	4.3606	1995	4.7746	4.1487	2006	3.8152	3.378
1985	4.0817	3.5541	1996	4.8266	4.2094	2007	—	—
1986	3.8653	3.4935	1997	4.8647	4.0579	2008	3.8717	3.2851
1987	4.5069	4.2427	1998	4.8128	3.9335	2009	3.9236	3.5933
1988	4.0626	3.7496	1999	4.7943	3.806	2010	3.202	2.4362
1989	4.3759	4.1305	2000	5.0238	3.5641			
1990	4.1538	3.8127	2001	—	—

### Estimation of Parameters

In order to carry out the numerical simulations, we need to estimate the model parameters. We obtain these parameter values using two approaches: some parameter values are adapted from literature; and some other parameter values are estimated by the MATLAB tool *fminsearch*, which is estimated by calculating the minimum sum of square (MSS):

$$MSS=\sum _{i=1}^{3}\,(\sum _{{n}_{i}}^{{N}_{i}}\,({({\mathrm{log}}_{10}(E({\text{data}}_{i}))-{\mathrm{log}}_{10}(E(i)))}^{2}+{({\mathrm{log}}_{10}(I({\text{data}}_{i}))-{\mathrm{log}}_{10}(I(i)))}^{2})),$$

where *n*_*i*_ = 1980, 2005, 2008, *N*_*i*_ = 2000, 2006, 2010, *i* = 1, 2, 3. All parameter values for Foshan City are given in Table 1. Next, we explain the parameter values as follows: (a) we fixed the natural death rates of humans and snails as ${\mu }_{h}=\frac{1}{72}$, ${\mu }_{s}=\frac{1}{1}$, ${\mu }_{g}=\frac{1}{26}$ and ${\mu }_{c}=\frac{365}{140}$, respectively, from the assumption that the average life lengths of humans, snails, eggs and cercariae are about 72 years^26^, 1 year^24,31^, 26 years^3^, and 140 days^3^, respectively. For infected human, the egg-laying capacity is estimated at around 4000 eggs per worm per day^3^, then we can estimate *P*_*g*_ = 4000 × 365 = 1.46 × 10^6^/*year*. Per capita recovery rate of human hosts is *γ* = 0.73^32^. We obtained *E*_*h*_(0) = 2882, *I*_*h*_(0) = 790 from^29^ and *R*_*h*_(0) = *I*_*h*_(0) × *γ* = 568. (b) Λ_*h*_, Λ_*s*_, Λ_*f*_ and other initial values, which are shown in Table 3, were regarded as parameters. The transmission rates *β*_*h*_ and *β*_*f*_, the released rate of cercaria from every infected snail *θ*_*c*_ are obtained by fitting in simulations and the same as *r*, *p* + *μ*_*f*_, Λ_*h*_, Λ_*s*_, Λ_*f*_. By the parameter values in Table 1, we can estimate that the basic reproduction number of human clonorchiasis is *R*_0_ = 2.01.

**Table 3:** Initial conditions (INC) of system (1).

INC	Value	Source	INC	Value	Source
*S*_*h*_(0)	1.9 × 10^5^	fitting	*S*_*s*_(0)	1000	fitting
*E*_*h*_(0)	2882	^29^	*I*_*s*_(0)	12	fitting
*I*_*h*_(0)	790	^29^	*C*(0)	14	fitting
*R*_*h*_(0)	568	^29, 32^	*S*_*f*_(0)	9.99 × 10^6^	fitting
*G*(0)	25	fitting	*I*_*f*_(0)	1.9 × 10^5^	fitting

### Applications to the *C*. *sinensis* infections in Foshan City

**Figure 3:**
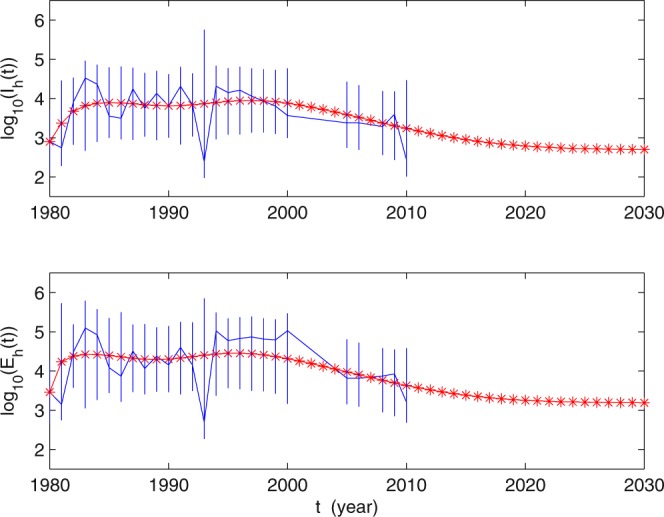
The tendencies of the values log_10_ (*E*(*t*)) and log_10_ (*I*(*t*)).

Using these parameter values, we carry out numerical simulations of our model and obtain a reasonable match in Fig. 2, indicating that our model provides a good match to the reported data. We would like to mention that from 1990–2010, integrated control strategies, including environmental management, repeated examination, education and capacity building through intersectoral collaboration, were advocated in Guangdong Province^29^. The awareness of the liver fluke disease for people from 1990 has been enhanced gradually, especially from 1994–2000. Particularly, inspection work in Collective-Owned group was carried out in Foshan City from 1997–2000^29^. This may explain why the number of infectious persons decreased and more people were inspected than in the previous years from 1994–2000. The model does not include these measures. While when the number of infectious humans is decreasing, people may not have the consciousness of this disease, so the number of inspected people may decrease again from 2000. This demonstrates further that our model has certain rationality. Figure 3 presents the tendency of clonorchiasis disease epidemics under the current control strategies and of the 95% confidence intervals. The result shows that the number of human clonorchiasis cases will decrease steadily in the future and finally becomes stable. This means that if no further effective prevention and control measures are taken, the disease will be epidemic in Foshan City.

### Sensitivity analysis

**Figure 4:**
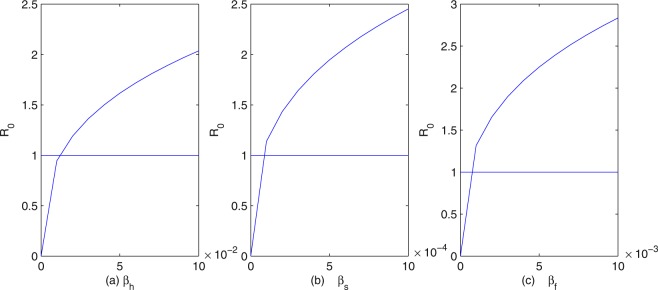
The dependence of *R*_0_ on (**a**) *β*_*h*_; (**b**) *β*_*s*_; (**c**) *β*_*f*_.

To provide some effective control measures about clonorchiasis, we perform some sensitivity analysis of the basic reproduction number *R*_0_ in terms of our model parameters. From Fig. 4, we can see that the influence of fish on *R*_0_ is greater than humans and snails. In fact, if we fix all the parameters except *β*_*s*_, *β*_*f*_, *β*_*h*_, then *R*_0_ increases as any of the transmission coefficients increases. However, *R*_0_ increases more rapidly as the transmission coefficient from cercariae to fish *β*_*f*_ increases than from fish to humans *β*_*h*_ and from eggs to snails *β*_*s*_. Thus, we know that the transmission of clonorchiasis from cercariae to fish plays a more important role than that from eggs to snails and from fish to humans. This strongly suggests that a more comprehensive approach needs to include environmental modification in order to break the cercaria-fish transmission cycle. In Fig. 4(a), *R*_0_ increases as *β*_*h*_ increases. Hence, the practical measure for preventing and controlling human infection is to reduce and stop the consumption of undercooked, freshly pickled or raw fish and shrimp flesh, making a decrease of *β*_*h*_. There is evidence showing that human beings can become infected via the accidental ingestion of *C*. *sinensis* metacercariae via their hands, contaminated as a consequence of not washing after catching freshwater fish^7^, then more attention should be paid to the safety of freshwater fish^17^. In addition, metacercaria-tainted fish should be barred from markets.

**Figure 5:**
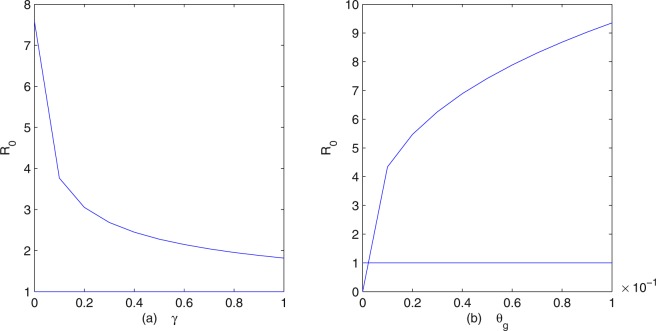
The dependence of *R*_0_ on (**a**) *γ*; (**b**) *θ*_*g*_.

Figure 5(a) shows the dependence of the basic reproduction number *R*_0_ on the recovery rate of human hosts *γ*, indicating that *R*_0_ decreases as *γ* increases. The disease cannot be eliminated even if *γ* = 1, which indicates that treatment with drugs alone is insufficient to achieve the complete control of clonorchiasis. As a matter of fact, residents in the epidemic areas find it is difficult to change their habit of eating raw fish and they have more opportunities to ingest food containing raw fish. For instance, in south China (for example Guangdong) and parts of east Asia, various species of carp, particularly *C*. *idellus* (grass carp), eaten raw as a “yusheng zhou” or as a “sushi”-fish congee, dipped in hot rice soup, are considered delicacies^9^. The sustainability of achievements in the long run is challenging, as the cycle of infection-treatment-reinfection continues, especially in the older age groups^33^. From Fig. 5(b) we see that *R*_0_ increases as the rate of eggs into the fresh water (snails) *θ*_*g*_ increases. This means that environmental modification is an important method of controlling clonorchiasis, such as removing unimproved lavatories built adjacent to fish ponds in endemic areas, thus preventing water contamination by faeces^9,11^. Removing pigsties and toilets from fishpond areas is an important step to decrease the source of eggs^17^, which is helpful in the field of environmental reconstruction. Furthermore, it is strongly necessary to inform farmers not to use human faeces as fertilizer, this breeding and cultivation practice can increase the risk of clonorchiasis infection because the faeces are highly saturated with *C*. *sinensis* eggs^1^.

## Discussion

Recognized as a neglected tropical disease by the World Health Organization for decades, clonorchiasis remains prevalent worldwide, although control programmes and some chemotherapy have been implemented over several years in some endemic areas. Clinical and epidemiological research into clonorchiasis over the past 140 years has contributed to a deeper understanding of the parasite, intermediate hosts, and disease^3^. Many interesting articles have also been published to investigate the prevention and control measures of the diseases, see^6,18,19,24,26^. Most of these studies focus on the pathology, biology, the discovery of new diagnostic, drug, and vaccine targets. Until now there is no study using mathematical models to assess different tools and strategies for large-scale control of clonorchiasis.

In this paper, we have proposed a deterministic model to describe the human-snail-fish transmission of clonorchiasis and studied its dynamical behavior. Meanwhile, our model can help in examining the current control and prevention policies. By estimating the parameter values, we obtianed *R*_0_ = 2.01, used the model to simulate the human clonorchiasis data from Foshan City reported in^29^, and predicted the spread of the disease in the city for the near future. We believe that it is the first time the human clonorchiasis data from Foshan City have been systematically simulated by using mathematical models. These numerical simulations indicate that the clonorchiasis disease has not reached its equilibrium yet and will become endemic in the future, which means that current control and prevention strategies cannot guarantee the eradication of the disease.

In order to find out effective control measures to prevent outbreaks of clonorchiasis in Foshan City, we performed various numerical simulations of our model. Figure 4 suggests that, to control and eventually eradicate clonorchiasis, a more comprehensive approach needs to include environmental factors^3^ in order to break the cercaria-fish transmission cycle. The infection rates and distributions of freshwater fish and snails should be investigated in endemic areas. These control measures include more comprehensive surveillance on fish, early check and vaccination of fish, and snail control by means of environment management. Indeed, an oral vaccine based on B subtilis expressing enolase is under test in freshwater fish^34^. Biological control, with predator fish that feed on snails, needs further investigation^35^. Figure 5(a) indicates that only by treatment with drugs cannot control and eventually eradicate *C*. *sinensis*. Today, praziquantel is the recommended drug of choice and tribendimidine might be an alternative^36^. But, cure rates were low, especially in the treatment of heavy infections^37^ and the cycle of infection-treatment-reinfection continues. Given the indirect economic losses and direct medical issues associated with clonorchiasis infection, there is a need for multifaceted prevention programs in addition to treatment with drugs. However, no commercially produced or effective vaccine is available for the treatment of clonorchiasis infection in humans or other hosts as of yet^17^. Researchers have obtained some protective actions, but only in rat models^21,23^.

There are some limitations in our study. Firstly, host heterogeneity was not included in the model, while different human groups may have different transmission patterns and different infection rates^38^. For example, males certainly have a higher infection rate than females. Secondly, the data we used were limited. Third, piscivorous animals, especially dogs and cats (both reared or wild as guardians or pets), serve as reservoir hosts for *C*. *sinensis*, and these animals are widely distributed^34,39^, but were not considered in this paper.

In conclusion, a combination of control strategies consisted of education, information and communication, treatment, environmental management, and preventive chemotherapy should be advocated for controlling the disease and preventing large local outbreaks.

## Electronic supplementary material

Supplementary material
