# Supplementary material for: Modeling the Transmission Dynamics of Clonorchiasis in Foshan, China
Source: Sci Rep. 2018 Oct 11;8:15176. doi: 10.1038/s41598-018-33431-w (PMC6181966; doi:10.1038/s41598-018-33431-w)
Supplement: Supplementary file 1 — Supplementary material [file 41598_2018_33431_MOESM1_ESM.pdf]

Supplementary Material

Modeling the Transmission Dynamics of Clonorchiasis in Foshan, China

Ruixia Yuan, Jicai Huang, Xinan Zhang and Shigui Ruan

S.1. Invariance properties

Let

$$\Omega = \{(S_h(t), E_h(t), I_h(t), R_h(t), G(t), S_s(t), I_s(t), C(t), S_f(t), I_f(t)) \in \mathbb{R}_+^{10} \mid$$
$$0 \le S_h(t) + E_h(t) + I_h(t) + R_h(t) \le \frac{\Lambda_h}{\mu_h},\ 0 \le S_s(t) + I_s(t) \le \frac{\Lambda_s}{\mu_s}, 0 \le S_f(t) + I_f(t) \le \frac{\Lambda_f}{p + \mu_f},$$
$$0 \le G(t) \le \frac{\theta_g P_g \Lambda_h}{\mu_g \mu_h},\ 0 \le C(t) \le \frac{\theta_c P_c \Lambda_s}{\mu_c \mu_s}\},$$

then, we can show that Ω is positively invariant for system (1).

Lemma S.1. Ω is a positively invariant set of system (1).
Proof. From the first four equations in model (1) we have

$$N_h'(t) = \Lambda_h - \mu_h N_h(t).$$

This implies that $N_h(t) \to \frac{\Lambda_h}{\mu_h}$ as $t \to +\infty$, so the limiting set of system (1) is on the plane $S_h(t) + E_h(t) + I_h(t) + R_h(t) = \frac{\Lambda_h}{\mu_h}$. Similarly, we can obtain

$$N_s'(t) = \Lambda_s - \mu_s N_s(t),$$
$$N_f'(t) = \Lambda_f - (p + \mu_f) N_f(t),$$

which means that the limiting set of system (1) is on the planes $S_s(t)+I_s(t) = \frac{\Lambda_s}{\mu_s}$ and $S_f(t)+I_f(t) = \frac{\Lambda_f}{p+\mu_f}$. These lead to

$$G'(t) \le \theta_g P_g \frac{\Lambda_h}{\mu_h} - \mu_g G(t),$$
$$C'(t) \le \theta_c P_c \frac{\Lambda_s}{\mu_s} - \mu_c C(t),$$

from this we obtain that $G(t) \le \frac{\theta_g P_g \Lambda_h}{\mu_g \mu_h}$ and $C(t) \le \frac{\theta_c P_c \Lambda_s}{\mu_c \mu_s}$. This proves the lemma.

S.2. Disease-free equilibrium and the basic reproduction number

Model (1) has a disease-free equilibrium given by

$$E^0 = (\frac{\Lambda_h}{\mu_h}, 0, 0, 0, 0, \frac{\Lambda_s}{\mu_s}, 0, 0, \frac{\Lambda_f}{p + \mu_f}, 0).$$

Next, we calculate the basic reproduction number R_0 and discuss the locally stability of the disease-free equilibrium in terms of R_0.

We order the infected variables first by disease state, only need to consider the vector $x = (E_h, I_h, I_s, I_f, G, C)^T$. Considering the following auxiliary system:

$$\begin{cases} E_h'(t) = \frac{\beta_h S_h(t) I_f(t)}{N_h(t)} - r E_h(t) - \mu_h E_h(t), \\ I_h'(t) = r E_h(t) - \gamma I_h(t) - \mu_h I_h(t), \\ I_s'(t) = \frac{\beta_s S_s(t) G(t)}{N_s(t)} - \mu_s I_s(t), \\ I_f'(t) = \frac{\beta_f S_f(t) C(t)}{N_f(t)} - (p + \mu_f) I_f(t), \\ G'(t) = \theta_g P_g I_h(t) - \mu_g G(t), \\ C'(t) = \theta_c P_c I_s(t) - \mu_c C(t). \end{cases} \quad (S1)$$

Model (S1) has a disease-free equilibrium given by $\overline{x_0} = (0,0,0,0,0,0)$. All solutions of system (S1) remain nonnegative and

$$\Omega_0 = \{(E_h(t), I_h(t), I_s(t), I_f(t), G(t), C(t)) \in \mathbb{R}_+^6 \mid 0 \le E_h(t) \le \frac{\Lambda_h}{\mu_h}, 0 \le I_h(t) \le \frac{\Lambda_h}{\mu_h}, 0 \le I_s(t) \le \frac{\Lambda_s}{\mu_s},$$
$$0 \le I_f(t) \le \frac{\Lambda_f}{p+\mu_f},\ 0 \le G(t) \le \frac{\theta_g P_g \Lambda_h}{\mu_g \mu_h},\ 0 \le C(t) \le \frac{\theta_c P_c \Lambda_s}{\mu_c \mu_s}\}$$

is positively invariant for system (S1).

We follow the recipe from van den Driessche and Watmough [1] to obtain

$$\mathcal{F}(x) = \begin{pmatrix} \frac{\beta_h S_h(t) I_f(t)}{N_h(t)} \\ r E_h(t) \\ \frac{\beta_s S_s(t) G(t)}{N_s(t)} \\ \frac{\beta_f S_f(t) C(t)}{N_f(t)} \\ 0 \\ 0 \end{pmatrix}, \mathcal{V}^-(x) = \begin{pmatrix} (r+\mu_h)E_h(t) \\ (\mu_h+\gamma)I_h(t) \\ \mu_s I_s(t) \\ (p+\mu_f)I_f(t) \\ \mu_g G(t) \\ \mu_c C(t) \end{pmatrix}, \mathcal{V}^+(x) = \begin{pmatrix} 0 \\ 0 \\ 0 \\ 0 \\ \theta_g P_g I_h(t) \\ \theta_c P_c I_s(t) \end{pmatrix}.$$

Therefore, system (S1) is equivalent to the following form

$$x' = \mathcal{F}(x) - \mathcal{V}(x), \tag{S2}$$

where $\mathcal{V}(x) = \mathcal{V}^-(x) - \mathcal{V}^+(x)$, which denotes the transfer rate of individuals into or out of each population set, and $\mathcal{F}(x)$ denotes the rate of occurrence of new infections. The next generation matrix is defined as FV^{-1}, F, V and V^{-1} are 6×6 Jacobian matrices given by

$$F = \begin{pmatrix} 0 & 0 & 0 & \beta_h & 0 & 0 \\ \mathrm{r} & 0 & 0 & 0 & 0 & 0 \\ 0 & 0 & 0 & 0 & \beta_s & 0 \\ 0 & 0 & 0 & 0 & 0 & \beta_f \\ 0 & 0 & 0 & 0 & 0 & 0 \\ 0 & 0 & 0 & 0 & 0 & 0 \end{pmatrix},$$

$$V = \begin{pmatrix} \mu_h + r & 0 & 0 & 0 & 0 & 0 \\ 0 & \mu_h + \gamma & 0 & 0 & 0 & 0 \\ 0 & 0 & \mu_s & 0 & 0 & 0 \\ 0 & 0 & 0 & (p+\mu_f) & 0 & 0 \\ 0 & -\theta_g P_g & 0 & 0 & \mu_g & 0 \\ 0 & 0 & -\theta_c P_c & 0 & 0 & \mu_c \end{pmatrix}.$$

The basic reproduction number R_0 is defined as the spectral radius of the nonnegative matrix FV^{-1}, so we obtain that

$$R_0 = \rho(FV^{-1}) = \sqrt[3]{\frac{\beta_h \beta_s \beta_f \theta_c P_c \theta_g P_g r}{\mu_s (p+\mu_f) \mu_c \mu_g (\mu_h+\gamma)(\mu_h+r)}}.$$

To discuss the properties of the disease free equilibrium E^0, we make an elementary row-

transformation for the Jacobian matrix at E^0 and obtain the following matrix:

$$J = \begin{pmatrix} -\mu_h & -(r+\mu_h) & 0 & 0 & 0 & 0 & 0 & 0 & 0 & 0 \\ 0 & -(r+\mu_h) & 0 & 0 & 0 & 0 & 0 & 0 & 0 & \beta_h \\ 0 & 0 & -(\gamma+\mu_h) & 0 & 0 & 0 & 0 & 0 & 0 & H_1 \\ 0 & 0 & 0 & -\mu_h & 0 & 0 & 0 & 0 & 0 & H_2 \\ 0 & 0 & 0 & 0 & -\mu_g & 0 & 0 & 0 & 0 & H_3 \\ 0 & 0 & 0 & 0 & 0 & -\mu_s & 0 & 0 & 0 & H_4 \\ 0 & 0 & 0 & 0 & 0 & 0 & -1 & 0 & 0 & H_5 \\ 0 & 0 & 0 & 0 & 0 & 0 & 0 & -\mu_c & 0 & H_6 \\ 0 & 0 & 0 & 0 & 0 & 0 & 0 & 0 & -(p+\mu_f) & H_7 \\ 0 & 0 & 0 & 0 & 0 & 0 & 0 & 0 & 0 & H_8 \end{pmatrix},$$

where

$$H_1 = \frac{\beta_h r}{r+\mu_h},\ H_2 = \frac{\beta_h r\gamma}{(r+\mu_h)(\gamma+\mu_h)},\ H_3 = \frac{\beta_h\theta_g P_g r}{(r+\mu_h)(\gamma+\mu_h)}, H_4 = -\frac{\beta_h\theta_g P_g r}{(r+\mu_h)(\gamma+\mu_h)}\frac{\beta_s}{\mu_g},$$

$$H_5 = \frac{\beta_h\theta_g P_g r}{(r+\mu_h)(\gamma+\mu_h)}\frac{\beta_s}{\mu_g}\frac{1}{\mu_s}, H_6 = \frac{\beta_h\theta_g P_g r}{(r+\mu_h)(\gamma+\mu_h)}\frac{\beta_s}{\mu_g}\frac{\theta_c P_c}{\mu_s},\ H_7 = -\frac{\beta_h\theta_g P_g r}{(r+\mu_h)(\gamma+\mu_h)}\frac{\beta_s}{\mu_g}\frac{\theta_c P_c}{\mu_s}\frac{\beta_f}{\mu_c},$$

$$H_8 = \frac{\beta_h\beta_s\beta_f\theta_c P_c\theta_g P_g r}{(r+\mu_h)\mu_s(p+\mu_f)\mu_c\mu_g(\mu_h+\gamma)} - 1 = R_0^3 - 1.$$

The eigenvalues are $\lambda_1 = \lambda_4 = -\mu_h < 0,\ \lambda_2 = -(r+\mu_h) < 0,\ \lambda_3 = -(\gamma+\mu_h) < 0,\ \lambda_5 = -\mu_g < 0,\ \lambda_6 = -\mu_s < 0,\ \lambda_7 = -1 < 0,\ \lambda_8 = -\mu_c < 0,\ \lambda_9 = -(p+\mu_f) < 0,\ \lambda_{10} = H_8$.

Then, $\lambda_{10} < 0$ if and only if

$$R_0 < 1.$$

Therefore, we obtain the following theorem.

Theorem S.2. The disease-free equilibrium E^0 of system (1) is locally asymptotically stable if $R_0 < 1$, and E^0 is unstable if $R_0 \geq 1$ in the region Ω.

S.3. Endemic equilibrium

Let $E^* = (S_h^*, E_h^*, I_h^*, R_h^*, G^*, S_s^*, I_s^*, C^*, S_f^*, I_f^*)$ denotes the endemic equilibrium. Here S_h^*, E_h^*,

I_h^*, R_h^*, G^*, S_s^*, I_s^*, C^*, S_f^*, I_f^* satisfy the following algebraic equations:

$$\begin{cases} \Lambda_h = \dfrac{\beta_h S_h^* I_f^*}{N_h^*} + \mu_h S_h^*, \\ \dfrac{\beta_h S_h^* I_f^*}{N_h^*} = rE_h^* + \mu_h E_h^*, \\ rE_h^* = \gamma I_h^* + \mu_h I_h^*, \\ \mu_h R_h^* = \gamma I_h^*, \\ \mu_g G^* = \theta_g P_g I_h^*, \\ \Lambda_s = \dfrac{\beta_s S_s^* G^*}{N_s^*} + \mu_s S_s^*, \\ \mu_s I_s^* = \dfrac{\beta_s S_s^* M^*}{N_s^*}, \\ \mu_c C^* = \theta_c P_c I_s^*, \\ \Lambda_f = \dfrac{\beta_f S_f^* C^*}{N_f^*} + (p+\mu_f) S_f^*, \\ (p+\mu_f) I_f^* = \dfrac{\beta_f S_f^* C^*}{N_f^*}. \end{cases} \tag{S3}$$

Direct calculation of system (S3) shows that system (1) admits a unique endemic equilibrium E^* in the interior of the feasible region Ω if $R_0 > 1$, where

$$I_f^* = \frac{\mu_s \mu_g \mu_c \Lambda_h \Lambda_s \Lambda_f (\gamma+\mu_h)(r+\mu_h)(R_0^3 - 1)}{\mu_s \mu_g \mu_c \beta_h \Lambda_s \Lambda_f (\gamma+\mu_h)(r+\mu_h) + \theta_g P_g \Lambda_h r \beta_h \beta_s (\beta_f \Lambda_s \theta_c P_c + \mu_s \mu_c \Lambda_f)},\ C^* = \frac{(p+\mu_f)\Lambda_f I_f^*}{\beta_f(\Lambda_f - (p+\mu_f) I_f^*)},$$

$$S_h^* = \frac{\Lambda_h^2}{\mu_h \Lambda_h + \mu_h \beta_h I_f^*}, E_h^* = \frac{\mu_h \beta_h I_f^* S_h^*}{\Lambda_h (r+\mu_h)},\ I_s^* = \frac{\mu_c (p+\mu_f)\Lambda_f I_f^*}{\theta_c P_c \beta_f (\Lambda_f - (p+\mu_f) I_f^*)},\ N_f^* = \frac{\Lambda_f}{(p+\mu_f)},\ N_h^* = \frac{\Lambda_h}{\mu_h},$$

$$I_h^* = N_h^* - (S_h^* + R_h^* + E_h^*),\ R_h^* = \frac{\gamma}{\mu_h} I_h^*,\ G^* = \frac{\mu_s \Lambda_s I_s^*}{\beta_s(\Lambda_s - \mu_s I_s^*)},\ S_f^* = N_f^* - I_f^*,\ N_s^* = \frac{\Lambda_s}{\mu_s},\ S_s^* = N_s^* - I_s^*.$$

To discuss the properties of the endemic equilibrium E^*, we make an elementary row-transformation for the Jacobian matrix at E^* and obtain the following matrix:

$$J = \begin{pmatrix} -\mu_h & -(r+\mu_h) & 0 & 0 & 0 & 0 & 0 & 0 & 0 & 0 \\ 0 & J_0 & 0 & 0 & 0 & 0 & 0 & 0 & 0 & \frac{\beta_h S_h^*}{N_h^*} \\ 0 & 0 & -(\gamma+\mu_h) & 0 & 0 & 0 & 0 & 0 & 0 & J_1 \\ 0 & 0 & 0 & -\mu_h & 0 & 0 & 0 & 0 & 0 & J_2 \\ 0 & 0 & 0 & 0 & -\mu_g & 0 & 0 & 0 & 0 & J_3 \\ 0 & 0 & 0 & 0 & 0 & -(\frac{\beta_s G^*}{N_s^*} + \mu_s) & 0 & 0 & 0 & J_4 \\ 0 & 0 & 0 & 0 & 0 & 0 & -1 & 0 & 0 & J_5 \\ 0 & 0 & 0 & 0 & 0 & 0 & 0 & -\mu_c & 0 & J_6 \\ 0 & 0 & 0 & 0 & 0 & 0 & 0 & 0 & -(\frac{\beta_f C^*}{N_f^*} + p + \mu_f) & J_7 \\ 0 & 0 & 0 & 0 & 0 & 0 & 0 & 0 & 0 & J_8 \end{pmatrix},$$

where

$$J_0 = -(r+\mu_h)(\frac{\beta_h I_f^*}{\mu_h N_h^*} + 1),$$

$$J_1 = \frac{\beta_h S_h^*}{N_h^*} \frac{r}{(r+\mu_h)(\frac{\beta_h I_f^*}{\mu_h N_h^*}+1)},$$

$$J_2 = \frac{\beta_h S_h^*}{N_h^*} \frac{r\gamma}{(r+\mu_h)(\gamma+\mu_h)(\frac{\beta_h I_f^*}{\mu_h N_h^*}+1)},$$

$$J_3 = \frac{\beta_h S_h^*}{N_h^*} \frac{r}{r+\mu_h} \frac{\theta_g P_g}{(\gamma+\mu_h)(\frac{\beta_h I_f^*}{\mu_h N_h^*}+1)},$$

$$J_4 = -\frac{\beta_h S_h^*}{N_h^*} \frac{r}{r+\mu_h} \frac{\theta_g P_g}{(\gamma+\mu_h)(\frac{\beta_h I_f^*}{\mu_h N_h^*}+1)} \frac{\beta_s S_s^*}{\mu_g N_s^*},$$

$$J_5 = \frac{\beta_h S_h^*}{N_h^*} \frac{r}{r+\mu_h} \frac{\theta_g P_g}{(\gamma+\mu_h)(\frac{\beta_h I_f^*}{\mu_h N_h^*}+1)} \frac{\beta_s S_s^*}{\mu_g N_s^*} \frac{1}{(\frac{\beta_s G^*}{N_s^*}+\mu_s)},$$

$$J_6 = \frac{\beta_h S_h^*}{N_h^*} \frac{r}{r+\mu_h} \frac{\theta_g P_g}{(\gamma+\mu_h)(\frac{\beta_h I_f^*}{\mu_h N_h^*}+1)} \frac{\beta_s S_s^*}{\mu_g N_s^*} \frac{\theta_c P_c}{(\frac{\beta_s G^*}{N_s^*}+\mu_s)},$$

$$J_7 = -\frac{\beta_h S_h^*}{N_h^*} \frac{r}{r+\mu_h} \frac{\theta_g P_g}{(\gamma+\mu_h)(\frac{\beta_h I_f^*}{\mu_h N_h^*}+1)} \frac{\beta_s S_s^*}{\mu_g N_s^*} \frac{\theta_c P_c}{(\frac{\beta_s G^*}{N_s^*}+\mu_s)} \frac{\beta_f S_f^*}{\mu_c N_f^*},$$

$$J_8 = \frac{\beta_h S_h^*}{N_h^*} \frac{r}{r+\mu_h} \frac{\theta_g P_g}{(\gamma+\mu_h)(\frac{\beta_h I_f^*}{\mu_h N_h^*}+1)} \frac{\beta_s S_s^*}{\mu_g N_s^*} \frac{\theta_c P_c}{(\frac{\beta_s G^*}{N_s^*}+\mu_s)} \frac{\beta_f S_f^*}{\mu_c N_f^*} \frac{1}{\frac{\beta_f C^*}{N_f^*}+\mu_f+p} - 1 = \frac{1}{R_0^3} - 1.$$

The eigenvalues are $\lambda_1 = \lambda_4 = -\mu_h < 0$, $\lambda_2 = -(r+\mu_h)(\frac{\beta_h I_f^*}{\mu_h N_h^*}+1) < 0$, $\lambda_3 = -(\gamma+\mu_h) < 0$, $\lambda_5 = -\mu_g < 0$, $\lambda_6 = -(\frac{\beta_s G^*}{N_s^*}+\mu_s) < 0$, $\lambda_7 = -1 < 0$, $\lambda_8 = -\mu_c < 0$, $\lambda_9 = -(\frac{\beta_f C^*}{N_f^*}+p+\mu_f) < 0$, $\lambda_{10} = J_8$.

Then, $\lambda_{10} < 0$ if and only if

$$R_0 > 1.$$

Theorem S.3. If $R_0 > 1$, there exits an unique endemic equilibrium E^* of system (1) and E^* is locally asymptotically stable in the region Ω when $R_0 > 1$.

References

[1] Van Den Driessche, P. & Watmough, J. Reproduction numbers and sub-threshold endemic equilibria for compartmental models of disease transmission. *Math. Biosci.* **180,** 29-48 (2002).
